# Secondary Effects of Glyphosate Action in *Phelipanche aegyptiaca*: Inhibition of Solute Transport from the Host Plant to the Parasite

**DOI:** 10.3389/fpls.2017.00255

**Published:** 2017-02-27

**Authors:** Tal Shilo, Baruch Rubin, Dina Plakhine, Shira Gal, Rachel Amir, Yael Hacham, Shmuel Wolf, Hanan Eizenberg

**Affiliations:** ^1^Department of Plant Pathology and Weed Research, Agricultural Research Organization, Newe Ya‘ar Research CenterRamat Yishay, Israel; ^2^The Robert H. Smith Faculty of Agriculture, Food and Environment, The Robert H. Smith Institute of Plant Sciences and Genetics, The Hebrew University of JerusalemRehovot, Israel; ^3^Department of Entomology, Agricultural Research Organization, Newe Ya‘ar Research CenterRamat Yishay, Israel; ^4^Migal Galilee Technology CenterKiryat Shmona, Israel

**Keywords:** Egyptian broomrape, *Orobanche*, germination, tomato, phloem, translocation, sugars

## Abstract

It is currently held that glyphosate efficiently controls the obligate holoparasite *Phelipanche aegyptiaca* (Egyptian broomrape) by inhibiting its endogenous shikimate pathway, thereby causing a deficiency in aromatic amino acids (AAA). While there is no argument regarding the shikimate pathway being the primary site of the herbicide's action, the fact that the parasite receives a constant supply of nutrients, including proteins and amino acids, from the host does not fit with an AAA deficiency. This apparent contradiction implies that glyphosate mechanism of action in *P. aegyptiaca* is probably more complex and does not end with the inhibition of the AAA biosynthetic pathway alone. A possible explanation would lie in a limitation of the translocation of solutes from the host as a secondary effect. We examined the following hypotheses: (a) glyphosate does not affects *P. aegyptiaca* during its independent phase and (b) glyphosate has a secondary effect on the ability of *P. aegyptiaca* to attract nutrients, limiting the translocation to the parasite. By using a glyphosate-resistant host plant expressing the “phloem-mobile” green fluorescent protein (GFP), it was shown that glyphosate interacts specifically with *P. aegyptiaca*, initiating a deceleration of GFP translocation to the parasite within 24 h of treatment. Additionally, changes in the entire sugars profile (together with that of other metabolites) of *P. aegyptiaca* were induced by glyphosate. In addition, glyphosate did not impair germination or seedling development of *P. aegyptiaca* but begun to exert its action only after the parasite has established a connection to the host vascular system and became exposed to the herbicide. Our findings thus indicate that glyphosate does indeed have a secondary effect in *P. aegyptiaca*, probably as a consequence of its primary target inhibition—via inhibition of the translocation of phloem-mobile solutes to the parasite, as was simulated by the mobile GFP. The observed disruption in the metabolism of major sugars that are abundant in *P. aegyptiaca* within 48 h after glyphosate treatment provides a possible explanation for this inhibition of translocation and might reflect a critical secondary effect of the herbicide's primary action that results in loss of the parasite's superior sink for solutes.

## Introduction

The obligate holoparasite Egyptian broomrape, *Phelipanche aegyptiaca* (Orobanchaceae, formerly known as *Orobanche aegyptiaca*), parasitizes dicotyledonous plants and causes severe yield loss in numerous vegetable crops (Eizenberg et al., 2012, 2013). The life cycle of *P. aegyptiaca*—like that of other members of the genus *Phelipanche*—can be roughly divided into two main phases (Joel, 2000): a short independent phase, starting from seed conditioning and continuing through germination to the establishment of a vascular connection to the host root, and a parasitic phase, which lasts throughout the rest of the parasite lifecycle. During the latter phase, the parasite depends entirely on the host plant for water, carbohydrates and nutrients (Heide-Jørgensen, 2013). The parasite attaches to the roots of its host by a haustorium, whose role is initially attachment to and penetration into the host root tissue (Joel and Losner-Goshen, 1994) and, later, the establishment of both xylem and phloem connections between the parasite and its host (Dörr and Kollmann, 1995; Joel, 2013). The haustorium has some functional similarities both to roots, in that it absorbs water and minerals, and to sink organs in that it attracts carbon molecules (Westwood, 2013). In the host-parasite system, organic carbon, nitrogen and some minerals are transported primarily via the phloem (Hibberd et al., 1999; Hibberd and Jeschke, 2001). Translocation to the parasite of small molecules, such as sugars, herbicides and possibly amino acids, also takes place, as has been previously described in some members of the genus *Orobanche* (Aber et al., 1983; Arjona-Berral et al., 1990; Nandula et al., 1999; Diaz-Sanchez et al., 2002). In addition, it is known that macromolecules are also transported in the host-parasite system. For example, viral DNA and RNA were transferred from infected tomato (*Solanum lycopersicum*) and tobacco (*Nicotiana tabacum*) plants to *P. aegyptiaca* (Gal-On et al., 2009), and movement of green fluorescent protein (GFP) was demonstrated between parasitized transgenic tomato plants, expressing GFP under the *At*SUC2 promoter (Imlau et al., 1999), and *P. aegyptiaca* (Aly et al., 2011).

The ability of plant sink tissues, such as fruits and roots, to import carbon is generally attributed (as was first posited by Ernst Münch, 1930) to the differential pressure caused by a gradient of sugars along the source-sink path and their consumption in the sink (Wang et al., 1993; Knoblauch et al., 2016). In *P. aegyptiaca*, it is, however, still not clear exactly what determines the parasite's sink strength and what allows it to overcome the sinks in the host plant. Some lines of evidence indicate that by accumulating high levels of mineral ions, sugars and sugar alcohols, holoparasites of the Orobanchaceae maintain a low water potential, which facilitates the uptake of water and nutrients, as has been observed in some algae and fungi (Harloff and Wegmann, 1993; Westwood, 2013; Hacham et al., 2016). The cleavage of the translocated sucrose to glucose and fructose by invertase (Aber et al., 1983; Fernández-Aparicio et al., 2016) is thought to contribute further to elevating the osmotic pressure and thereby to provide the driving force for translocation of assimilates to the parasite (Delavault et al., 2002; Abbes et al., 2009b). Another factor contributing to elevation of the osmotic pressure in *P. aegyptiaca* and some of its close relatives is believed to be high levels of the sugar alcohol mannitol (Aly et al., 2009). The entire mannitol biosynthetic pathway has been documented in *P. ramosa* and *O. crenata* (Harloff and Wegmann, 1993), as has the activity of the key enzyme mannose-6-phosphate reductase (M6PR; EC 1.1.1.224) in *P. ramosa* (Delavault et al., 2002). It seems that this pathway is of importance to parasite survival, especially since silencing of the parasite's *M6PR* gene, by using a siRNA construct, resulted in a reduction of mannitol levels concomitant with increased mortality of *P. aegyptiaca* (Aly et al., 2009).

Despite the considerable amount of work that has been devoted to *P. aegyptiaca*, this parasitic plant still poses a challenge in weed management, particularly because only a few herbicides are available for its control (Eizenberg et al., 2013). Among these, the non-selective herbicide glyphosate can efficiently control *P. aegyptiaca* (Joel et al., 1995), even in low doses (Jacobsohn and Kelman, 1980; Cochavi et al., 2015). Glyphosate inhibits the enzyme 5-enolpyruvyl shikimate-3-phosphate synthase (EPSPS; EC 2.5.1.19) (Steinrücken and Amrhein, 1980), a key enzyme in the shikimate pathway and in the aromatic amino acids (AAA) biosynthesis pathway. After absorption into the plant foliage, glyphosate is translocated mainly into the symplast and accumulates mostly in sink tissues (Gougler and Geiger, 1981; Schulz et al., 1990). Tubercles of *P. aegyptiaca* and of other close relatives in the genus have been shown to accumulate high amounts of [^14^C]glyphosate within 24 h after application of the herbicide to host plants (Arjona-Berral et al., 1990; Jain and Foy, 1997; Diaz-Sanchez et al., 2002). Furthermore, accumulation of shikimate has been detected in *P. aegyptiaca* as early as 10 h after treatment (HAT) with glyphosate (Shilo et al., 2016), indicating the presence of the herbicide in the parasite tissue. In addition to its movement in the plant, some evidence indicates that glyphosate is exuded from the roots of treated plants and also acts in the rhizosphere (Coupland and Caseley, 1979; Kremer et al., 2005; Laitinen et al., 2007). While it is clear that glyphosate reaches and acts in *P. aegyptiaca* tubercles, the fate of the parasite seeds and seedlings upon exposure to glyphosate in the host rhizosphere, before attachment occurs, remains unknown. While there is no argument that glyphosate inhibits specifically its target enzyme—EPSPS, the mode of action of glyphosate is thought to be more complex than the inhibition of the AAA biosynthetic pathway alone. The importance of the shikimate pathway in the metabolism of primary and secondary compounds implies that its inhibition might influence a variety of processes in the plant (Tzin and Galili, 2010). Each one of these processes, once inhibited, could potentially lead to death of the plant. On the one hand, in some autotrophic plants symptoms resulting from the application of glyphosate tend to develop slowly, in keeping with a deficiency of AAA and disruption of protein biosynthesis (Gresshoff, 1979; Duke and Powles, 2008). On the other hand, several secondary effects of glyphosate action have been described, including: (1) a decrease in the rate of photosynthesis and in concentrations of photosynthates in source tissues (Servaites et al., 1987; Geiger and Bestman, 1990; Fuchs et al., 2002); (2) inhibition of the biosynthesis of phenolic compounds, resulting in impaired defense mechanisms (Lévesque and Rahe, 1992); (3) interference with auxin levels, leading—in glyphosate resistant-cotton—to re-organization of the anther tissue and resultant male sterility (Yasuor et al., 2006); (4) injury to glyphosate-resistant-soybean possibly as a result of phytotoxicity of aminomethylphosphonic acid (AMPA), a metabolite of glyphosate (Reddy et al., 2004); (5) leaching of glyphosate along with other metabolites into the soil, resulting in improved pathogen success (Kremer et al., 2005); (6) limitation of water availability to the shoots (Fuchs et al., 2002); and (7) disruption of carbon metabolism, leading to limitation of transport within the plant, either of glyphosate itself (Geiger et al., 1999) or of carbon compounds (Geiger and Bestman, 1990; Geiger et al., 1999; Orcaray et al., 2010).

The inhibition of EPSPS causes a loss of feedback inhibition of 3-deoxy-d-arabinoheptulosonate 7-phosphate synthase (DAHPS; EC 2.5.1.54), an enzyme of the shikimate pathway, which leads to an extremely high accumulation of shikimate (Duke, 1988). Since the shikimate pathway metabolizes about 30% of the assimilated carbon in photosynthetic plants (Maeda and Dudareva, 2012), it is reasonable to assume that related metabolic pathways may be affected within hours after the inhibition of EPSPS, due to the impairment of carbon metabolism (Servaites et al., 1987; Siehl, 1997; Orcaray et al., 2012). These changes, in turn, might lead to changes in source-sink relations and limit the translocation of solutes, as seen in autotrophic plants and also in a study of *Cuscuta campestris* parasitism, in which the translocation of [^14^C]sucrose and GFP in the phloem were limited by glyphosate application to the host plants (Nadler-Hassar et al., 2004).

In *P. aegyptiaca*, levels of AAAs were significantly decreased within 48 h after application of glyphosate to a tomato host, which is indicative both of the presence of an active shikimate pathway in the parasite and implies of the dependence of the parasite on self-production of AAAs (Shilo et al., 2016). This finding of a metabolite deficiency in *P. aegyptiaca* is something of an enigma in light of its ability to strongly attract nutrients, including proteins (Aly et al., 2011) and free amino acids (Aber et al., 1983; Gaudin et al., 2014), from the host. We thus posit that glyphosate acts in *P. aegyptiaca* not only by creating a deficiency in AAA, but also through disruption of pathways that are associated with the shikimate pathway, causing the inhibition of transport of solutes from the host to the parasite.

The objective of the current study was thus to examine aspects of *P. aegyptiaca* control by glyphosate that are yet to be resolved, as follows: (a) during the independent life cycle phase, to examine the effect of the herbicide on seed germination, on the viability of the seedlings and the ability of the seedlings to establish the vital connection to the hosts' vascular system, and (b) during the parasitism phase, to examine the possibility of a secondary effect of glyphosate's primary mechanism acting through the inhibition of translocation of solutes from the host to the parasite.

## Materials and methods

### Plant material

Tomato plants (*Solanum lycopersicum* Mill., var. UC-82, kindly provided by “Hishtil,” Israel) were used as the glyphosate-sensitive host (GST). The tomato germplasm 1232 (F_4_ self-cross, Zygier, 2005) was used as a glyphosate-resistant host (GRT). A transgenic tomato expressing GFP in its companion cells under the *At*SUC2 promoter (Imlau et al., 1999) was used as a GFP host sensitive to glyphosate (GGST). In these plants, GFP is secreted into the phloem system and is transported throughout the plant along the source-to-sink pathway (Imlau et al., 1999; Nadler-Hassar et al., 2004; Birschwilks et al., 2006; Aly et al., 2011). A GFP host resistant to glyphosate (GGRT) was prepared by crossing GGST plants with GRT plants. The F_1_ hybrids (GGRT) were resistant to glyphosate and expressed GFP in the phloem.

*Phelipanche aegyptiaca* Pers. seeds were collected in a commercial processing tomato field in the Hula valley in Northern Israel and were surface sterilized by soaking in 70% ethanol for 3 min and then in 1% sodium hypochlorite for 10 min, followed by washing four times in sterile water, before being dried and stored at 4°C in the dark.

### Germination of *P. aegyptiaca*

Seeds of *P. aegyptiaca* were spread in petri dishes on GF/A filter paper (Whatman, GE healthcare, UK) discs soaked in sterile distilled water and left for pre-conditioning for 7 days in an incubator (21.5°C constant). Thereafter, a solution of the strigolactone-analog GR24 (30 μl; 10^−5^ M) was added to the filter paper discs. Glyphosate (30 μl) was applied to the petri dishes as described below, either at the beginning of the pre-conditioning period (for the herbicide to be taken up during imbibition) or 2 days after addition of GR24 (after the germination process had commenced). Germination was documented 7 days after addition of GR24.

### Experimental system of host-parasite association

Hosts and parasites were grown hydroponically in polyethylene bags (PEBs) in a growth chamber [20°C/25°C (night/day); 14 h light, 150–200 μE m^−2^ s^−1^] according to Parker and Dixon (1983), as modified by Eizenberg et al. (2003). Briefly, 4-week-old tomato seedlings were thoroughly washed free of soil, re-rooted and transferred to the center of a PEB containing half-strength Hoagland solution (Hoagland and Arnon, 1950) on GF/A filter paper. PEBs were kept inside a dark box to exclude light from the root zone. For sugars analysis, GRT were used as host plants. With the planting of GRT plants in the PEB, sterile *P. aegyptiaca* seeds were spread on the GF/A paper. GR24 solution (4 ml; 5 ppm) was injected into the PEB 10 days after planting.

**Figure 1:**
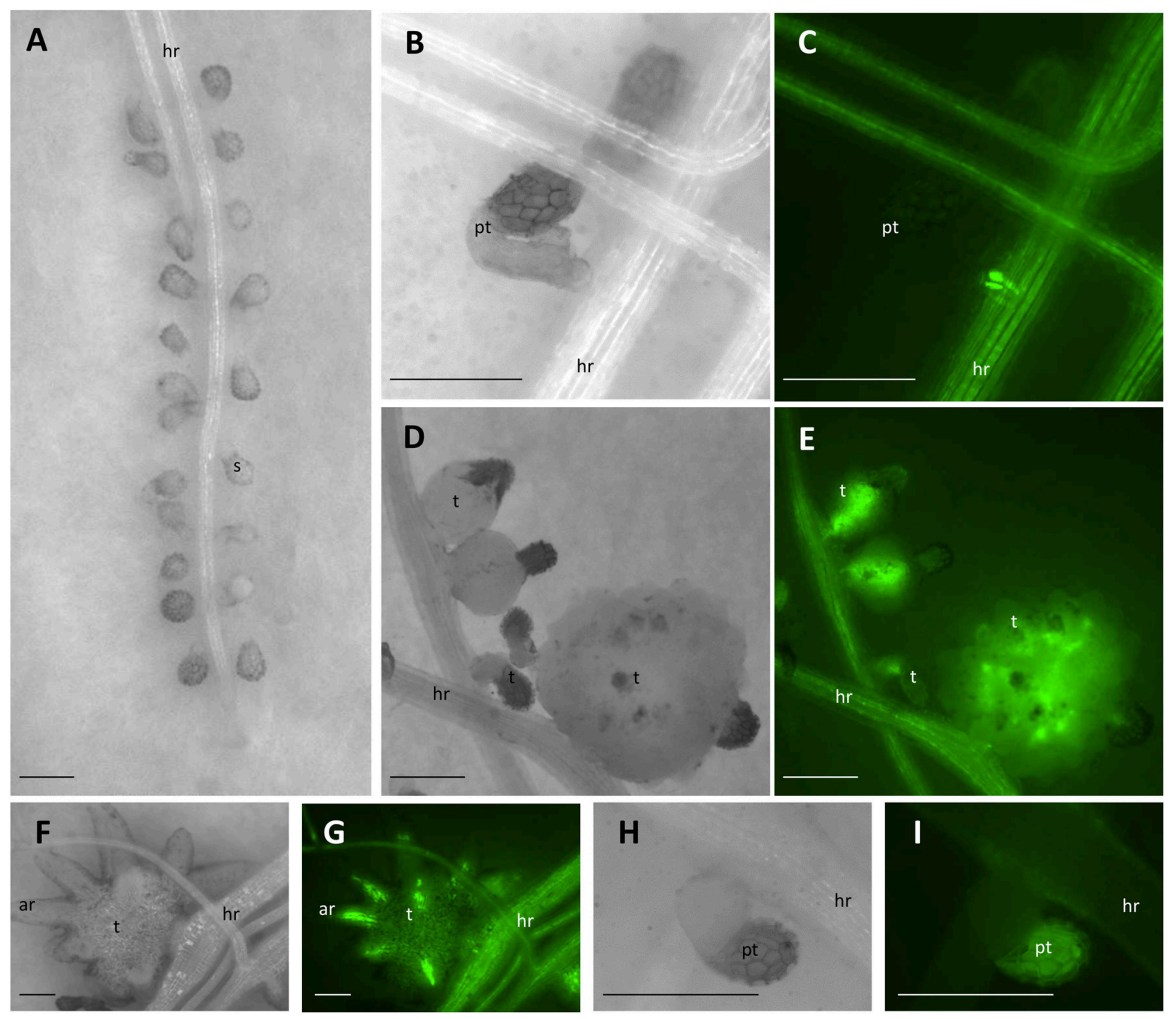
**Parasitism of *P. aegyptiaca* on the “GFP-mobile” host. (A)** Seeds of *P. aegyptiaca* were placed up to 0.5 mm from the root. **(B,C)** The parasite endophyte displaying fluorescence after forming a primary phloem connection. **(D,E)** Tubercles of *P. aegyptiaca* accumulating GFP. **(F,G)** “Spider” tubercle translocating GFP to its adventitious roots through the parasite phloem. **(H,I)** Attachment of *P. aegyptiaca* seed to a wild-type host. Notice the auto-fluorescence of the seed coat and the lack of fluorescence in the root of the host or in the tubercle. Images **(C,E,G,I)** were captured under GFP3 filter. hr, host root; s, seedling; pt, primary tubercle; t, tubercle; ar, adventitious root. Bar length represents 0.5 mm.

For GFP accumulation analysis, GGST and GGRT were used as host plants. Sterile *P. aegyptiaca* seeds were pre-conditioned in a petri dish as described above. Twenty four hours following the addition of GR24 (125 μl; 10^−5^ M), the seeds were transferred to the PEB in which they were placed close to randomly selected host roots at a distance not greater than 0.5 mm (Figure 1A). Germination of *P. aegyptiaca* occurred uniformly 3 days after the application of GR24, when the seeds were already in the PEBs; that day was recorded as the day of germination according to which all treatments were applied.

### Glyphosate application

For the germination analysis, glyphosate (Roundup®, 360 g ae l^−1^, Monsanto, USA) was applied in one of three doses: 18, 180, or 1,800 mg ae l^−1^. The highest dose was chosen to simulate an equivalent dose to 360 g ae ha^−1^ (in a spraying volume of 2,00l). For the GFP accumulation analysis, glyphosate was applied to the tomato plants grown in PEB either on the day of *P. aegyptiaca* germination or 7 days after germination (DAG) at a dose of 18 μg per plant on three leaves in 4 drops of 5 μl each. For the sugars analysis, glyphosate was similarly applied 5 weeks after planting in PEB.

### GFP accumulation analysis

GFP accumulation was monitored *in situ* in *P. aegyptiaca* parasitizing GGST and GGRT hosts from germination onwards for a period of 14 DAG or in a higher resolution experiment at smaller intervals for a period of 48 h. As soon as *P. aegyptiaca* penetrated the vascular system of a “GFP-mobile” host and connected to its phloem elements, intensified fluorescence was visible. In very early stages of the parasitism, the fluorescence was observed in the endophyte itself (Figures 1B,C), and later, in the forming tubercle (Figures 1D,E), including in the parasitic phloem system, once formed (Figures 1F,G). Although, auto-fluorescence of the seed coat of *P. aegyptiaca* was also detected (Figures 1H,I), it did not interfere with the observation and measurement of fluorescence in the tubercle itself. Images were captured using a fluorescent stereo microscope (Leica M205FA, Leica Microsystems GmbH, Germany), under a GFP3 filter (excitation: 450–490 nm, Leica Microsystems GmbH, Germany). The number of fluorescent tubercles was recorded as a measure of the progress of connection to the host phloem, and images were taken for subsequent measurement of the tubercles' area and fluorescence intensity by using ImageJ software (US National Institutes of Health, Bethesda, MD, USA, Schneider et al., 2012) according to Burgess et al. (2010) and Gavet and Pines (2010). Fluorescence intensity was quantified with the aim to assess the extent of translocation of the phloem solutes to the parasite, according to the following equation:

(1) $$\begin{array}{l} FI=ID-\left(TA\times MFB\right) \end{array}$$

where *FI*—the intensity of fluorescence, *ID*—the integrated density, *TA*—the area of the tubercle and *MFB*—the mean fluorescence of the background. Fluorescence intensity was also measured in untreated GST plants to eliminate the possibility of auto-fluorescence of the tissues.

### Sugars analysis

GRT hosts and parasites were grown in PEB and treated with glyphosate as described above. Tubercles and host roots (100 mg fresh weight) were sampled 48 HAT, frozen immediately in liquid nitrogen, and stored at −80°C until analysis.

Soluble carbohydrates were extracted from the above tissues and quantified according to Lisec et al. (2006) and Kazachkova et al. (2013) with minor modifications. Frozen samples were ground in 1 ml of extraction buffer containing HPLC-grade methanol:chloroform:distilled water (50:20:20 v:v:v). Norleucine (6.6 μl; 2 mg/ml) was added as an internal quantitative standard for the polar phase. Samples were then centrifuged (20,800 *g*) for 10 min at 4°C, and supernatants were transferred to new tubes. Distilled water (300 μl) and chloroform (300 μl) were added, and the vials were vortexed thoroughly and centrifuged (20,800 *g*) for 5 min at 4°C. Aliquots (300 μl) of the polar upper phase were transferred to new tubes and then dried in a vacuum concentrator (Centrivap concentrator, Labconco, Kansas city, MO, USA) overnight without heating and stored at −80°C until derivatization. For derivatization of the polar phase, frozen samples were dried again in vacuum concentrator for 30 min. To each tube, 40 μl of methoxyamine hydrochloride (20 mg/ml in pyridine) were added, and the mixture was shaken for 2 h at 37°C. Thereafter, 100 μl of MSTFA (N-methyl-N (trimethylsilyl)-trifluoroacetamide) and 7 μl of alkane standard mixture were added, and the mixture was shaken again for 30 min at 37°C. After a short spin, samples were transferred to GCMS auto-sampler vials.

Analysis was performed on a GCMS system (Agilent 7890A, Agilent Technologies, Inc., Santa Clara, CA, USA) coupled with a mass selective detector (Agilent 5975c, Agilent Technologies, Inc., Santa Clara, CA, USA) using a capillary VF-5ms column (Agilent Technologies, Inc., Santa Clara, CA, USA). Chromatograms and mass spectra were evaluated using MSD Chemstation (Agilent Technologies, Inc., Santa Clara, CA, USA). Sugars were identified and annotated in comparison with standards and with libraries of the National Institute of Standards and Technology (NIST) and the Max-Planck Institute for Plant Physiology Golm Metabolome Database (GMD). The peak area of each sugar metabolite was normalized according to the norleucine internal standard.

### Statistical analysis

Statistical analysis was carried out as described by Onofri et al. (2010). The experiments were arranged in a fully randomized design, with 3–6 replications as indicated in the figures. ANOVA and mean comparison were calculated by Student's *t*-test (α < 0.05) using JMP (version 5.1; SAS Inc., NC, USA). Non-linear regressions were computed by SigmaPlot (version 11.01; SPSS Inc., Chicago, IL, USA) and were used to describe the number of attachments and the accumulation of GFP in *P. aegyptiaca* and the response of these factors to glyphosate treatment according to the following equations:

(2) $$\begin{array}{l} \text{Sigmoid},3\text{ parameters}:\;y=\frac{a}{1+e^{-\left(\frac{x-x_{0}}{b}\right)}} \end{array}$$

where *x*—the time in DAG, *y*—the number of fluorescent tubercles or the intensity of fluorescence (as indicated in Tables 1, 2), *a*—the upper asymptote, *X*_*0*_—the inflection point, representing the time at 50% of the amplitude, and *b*—the slope at the inflection point.

(3) $$\begin{array}{l} \text{Gaussian},3\text{ parameters}:\;y=ae^{\left[-0.5{\left(\frac{x-x_{0}}{b}\right)}^{2}\right]} \end{array}$$

where *x*—the time in DAG, *y*—the number of fluorescent tubercles or the intensity of fluorescence (as indicated in Tables 1, 2), *a*—the amplitude, *X*_*0*_—the time at the peak point, and *b* controls the spread of the curve.

**Table 1:** **Regression coefficients and statistical analysis for Figure 4**.

**Figure**	**y Variable**	**Treatment**	**Regression**		**Parameters**			**Regression**	
					**Coefficient**	**SE**	***P***	***RMSE***	***P***
Figure 4B	No. of fluorescent tubercles (% of max)	Untreated	Sigmoid (Equation 2)	a	98.78	1.42	<0.0001	3.62	<0.0001
				b	1.13	0.09	<0.0001		
				X_0_	5.00	0.10	<0.0001		
		Glyphosate	Gaussian (Equation 3)	a	96.70	3.38	<0.0001	6.84	<0.0001
				b	2.12	0.08	<0.0001		
				X_0_	7.64	0.10	<0.0001		
Figure 4C	Tubercle area (mm^2^)	Untreated	Sigmoid (Equation 2)	a	2.01	0.32	0.0002	0.08	<0.0001
				b	2.32	0.41	0.0005		
				X_0_	10.86	0.88	<0.0001		
		Glyphosate	Sigmoid (Equation 2)	a	0.07	0.004	<0.0001	0.009	0.0011
				b	1.68	0.63	0.0259		
				X_0_	1.47	0.63	0.0465		
Figure 4D	Fluorescence intensity (arbitrary units)	Untreated	Sigmoid (Equation 2)	a	150.62	10.48	<0.0001	15.35	<0.0001
				b	1.19	0.25	0.0003		
				X_0_	8.49	0.32	<0.0001		
		Glyphosate	Gaussian (Equation 3)	a	2.69	0.40	<0.0001	0.85	0.0151
				b	3.16	0.65	0.0003		
				X_0_	7.02	0.55	<0.0001		

*Glyphosate was applied pre-attachment on the day of germination (0 DAG)*.

**Table 2:** **Regression coefficients and statistical analysis for Figure 5**.

**Figure**	**y Variable**	**Treatment**	**Regression**		**Parameters**			**Regression**	
					**Coefficient**	**SE**	***P***	***RMSE***	***P***
Figure 5B	No. of fluorescent tubercles (% of max)	Untreated	Sigmoid (Equation 2)	a	98.78	1.42	<0.0001	3.62	<0.0001
				b	1.13	0.09	<0.0001		
				X_0_	5.00	0.10	<0.0001		
		Glyphosate	Gaussian (Equation 3)	a	103.61	4.54	<0.0001	9.55	<0.0001
				b	2.67	0.14	<0.0001		
				X_0_	8.19	0.14	<0.0001		
Figure 5C	Tubercle area (mm^2^)	Untreated	Sigmoid (Equation 2)	a	2.01	0.32	0.0002	0.08	<0.0001
				b	2.32	0.41	0.0005		
				X_0_	10.86	0.88	<0.0001		
		Glyphosate	Sigmoid (Equation 2)	a	0.40	0.03	<0.0001	0.05	<0.0001
				b	1.63	0.56	0.0183		
				X_0_	5.13	0.58	<0.0001		
Figure 5D	Fluorescence intensity (arbitrary units)	Untreated	Sigmoid (Equation 2)	a	150.62	10.48	<0.0001	15.35	<0.0001
				b	1.19	0.25	0.0003		
				X_0_	8.49	0.32	<0.0001		
		Glyphosate	Gaussian (Equation 3)	a	39.40	2.90	<0.0001	6.85	<0.0001
				b	2.87	0.29	<0.0001		
				X_0_	9.11	0.27	<0.0001		

*Glyphosate was applied post-attachment, a week after germination (7 DAG)*.

Partial least square discriminant analysis (PLS-DA) was computed for the peak area measurements of the sugars using Metaboanalyst 3.0 (http://www.metaboanalyst.ca/) (Xia and Wishart, 2011; Xia et al., 2015) with auto scaling (mean-centered and divided by the standard deviation of each variable).

## Results

### The independent stage of *P. aegyptiaca*

#### Glyphosate has no effect on germination and seedling development of *P. aegyptiaca*

**Figure 2:**
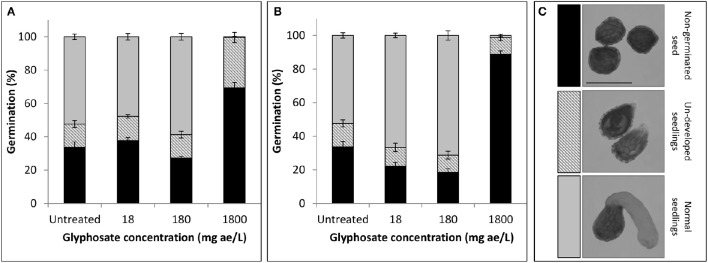
**Germination rate (%) of *P. aegyptiaca* seeds after treatment with glyphosate (A)** at the beginning of the pre-conditioning period or **(B)** 2 days after addition of GR24. Key—**(C)** Classification of seeds and seedlings corresponding to bar colors, as follows: non-germinated seeds (black); undeveloped seedlings (stripes); normal seedlings (gray). Error bars represent standard error (*n* = 4). Bar length represents 0.5 mm.

The fate of *P. aegyptiaca* seeds and seedlings exposed to glyphosate in the soil solution is not known, but it is relevant to the understanding whether glyphosate also prevents success of this parasite during its independent stage of life. In a petri dish experiment, it was found that under low doses of glyphosate (18 and 180 mg ae l^−1^) imbibed during the pre-conditioning period (Figure 2A) the portion of germinated glyphosate-treated seeds (for both doses) remained similar to that in the untreated group according to comparison of the means (*P* < 0.0001). When glyphosate was applied during the germination process (Figure 2B), the portion of germinated seeds was significantly higher for both doses, reaching 67% or 71% respectively, compared with 52% in the untreated group. For untreated seeds or seeds treated with low-dose glyphosate, similar portions of undeveloped seedlings were observed (Figure 2). These seedlings apparently started the process of germination but did not develop further, remaining spiky and sometimes necrotic. These results indicate that exposure of *P. aegyptiaca* during the independent growth stage to doses of glyphosate that may persist in the soil and are common in *P. aegyptiaca* management practice will basically not inhibit seed germination or the establishment of seedlings. In contrast, for the high dose of 1,800 mg ae l^−1^ glyphosate, the germination of *P. aegyptiaca* was almost completely inhibited, even though GR24 was added at the end of the pre-conditioning period. For both application times—at the beginning of the preconditioning period and 2 days after GR24 addition—the sum of ungerminated seeds plus nonviable seedlings totaled to more than 99 and 98%, respectively (Figures 2A,B).

#### Glyphosate has no effect on attachment of *P. aegyptiaca* to the host root

To examine the effect of glyphosate on the ability of *P. aegyptiaca* to attach to the host root, an *in-situ* experimental system was constructed to allow tracking of the process of attachment to the host vascular system by using GFP as a marker for the start of the translocation of solutes.

**Figure 3:**
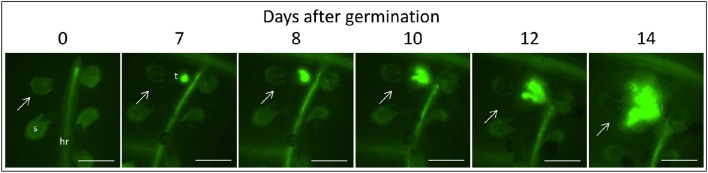
**Time course of attachment to untreated GGRT plants, captured under a GFP3 filter**. hr, host root; s, seedling; t, tubercle; Arrows indicate attachments. Bar length represents 0.5 mm.

**Figure 4:**
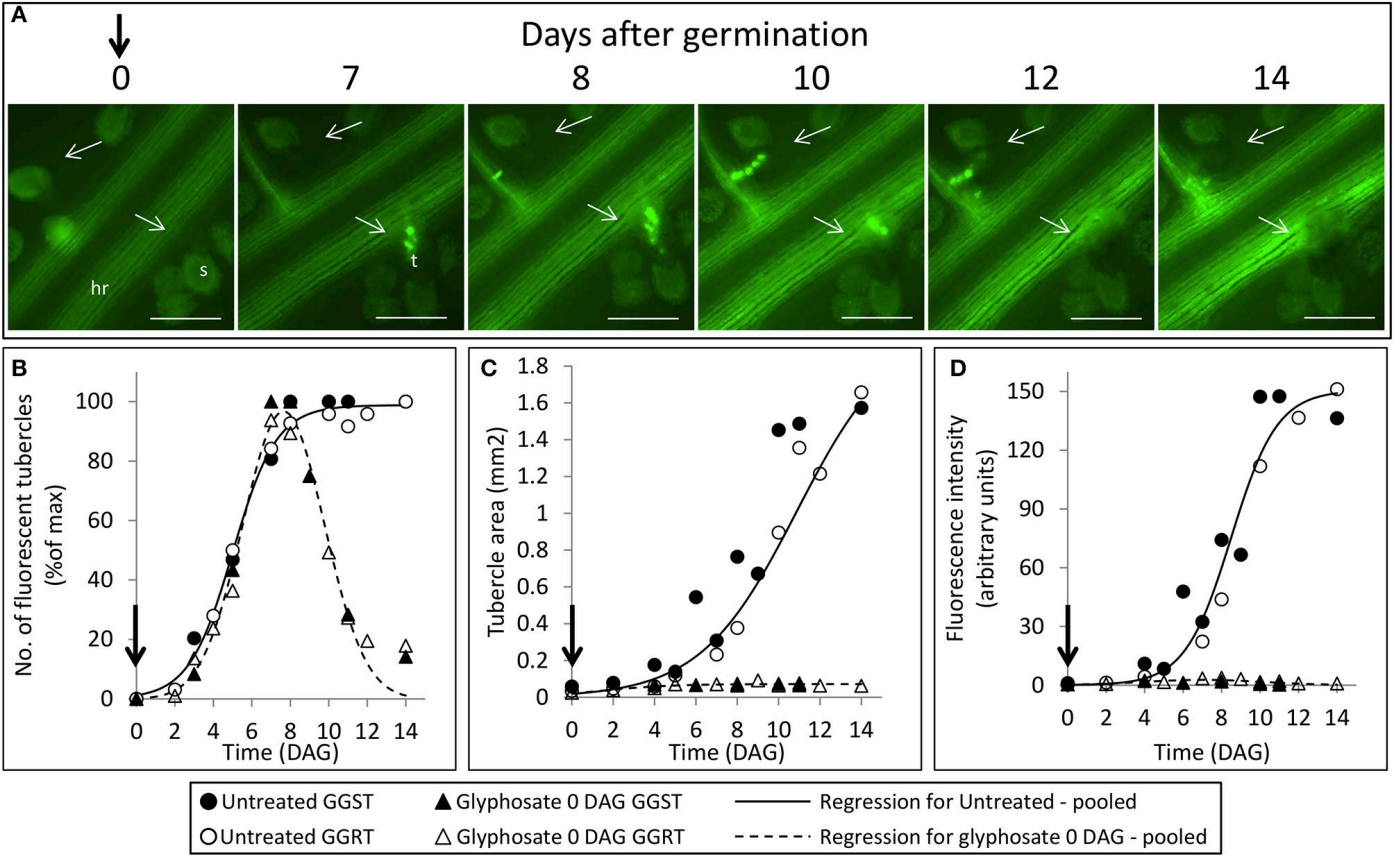
**Attachment and development of *P. aegyptiaca* following treatment with glyphosate at 0 DAG (pre-attachment). (A)** Time course of attachment to GGRT plants following treatment with glyphosate, captured under a GFP3 filter. hr, host root; s, seedling; t, tubercle; Arrows indicate attachments. Bar length represents 0.5 mm. **(B)** Number of active attachments (represented by the number of fluorescent tubercles), **(C)** area of the tubercle, and **(D)** accumulation of GFP in the tubercles (represented by the intensity of fluorescence) on GGST hosts (filled symbols) and on GGRT hosts (open symbols) following treatment with glyphosate at 0 DAG (triangles) or without treatment (circles). For non-linear regressions, data from GGST and GGRT hosts was subjected to the *F*-test and combined. DAG—days after germination. Regression parameters are presented in Table 1. (*n* = 6). Vertical arrows indicate the time of glyphosate application (0 DAG).

The count of fluorescent tubercles showed that connection to the phloem started as early as 2 DAG, while most of the connections occurred between 4 and 8 DAG (Figures 3, 4B). This pattern was similar for *P. aegyptiaca* parasitizing both GGST and GGRT hosts (*F*-test for regressions), and therefore the data for the two hosts were pooled (Figure 4B). When glyphosate was applied during the independent phase (pre-attachment), *P. aegyptiaca* seedlings were able to penetrate into the phloem similarly to the untreated group (Figure 4A), and first connections were similarly visible 2 DAG (Figure 4B). For both treated and untreated groups, most of the connections were established up to 7 DAG, but from that day onwards the number of fluorescent tubercles decreased in the treated groups (Figure 4B). A loss of GFP fluorescence and darkening occurred for each specific tubercle 3–4 days after the connection to the host (Figure 4A vs. Figure 3). These results indicate that *P. aegyptiaca* seedlings have the ability to attach normally, but once attached, their development is impaired by the herbicide. *P. aegyptiaca* responded similarly to glyphosate on both sensitive and resistant hosts (*F*-test for the regressions) and thus, here too, the data for the two hosts were pooled to create a single non-linear peak regression (Figure 4B). To normalize the number of attachments and established tubercles, the number of fluorescent tubercles was computed as percent of the maximal number (Figure 4B). However, it is important to note that *P. aegyptiaca* did successfully establish a phloem connection in the treated group to an extent similar to that for the untreated parasites (data not shown).

### The parasitic stage of *P. aegyptiaca*

#### Glyphosate hinders normal development of the young tubercle

Once connected to the vascular system of the host, *P. aegyptiaca* enters the parasitic life phase. In the early stages of the parasitism, the tubercles of *P. aegyptiaca* developed and increased in size (area) exponentially (Figure 4C). Additionally, proper development of the tubercle was indicated by its ability to attract solutes from the host phloem, as demonstrated by the level of fluorescence intensity, indicating accumulation of GFP (Figures 3, 4D). Accumulation of GFP started in the untreated tubercles at 4 DAG, while massive accumulation took place 7–10 DAG. The pattern of GFP transport in the phloem followed a sigmoidal trend and did not differ significantly between the two hosts (*F*-test) and therefore the data for the two hosts were pooled (Figure 4D). The maximal values of the measured fluorescent intensity—represented in the upper asymptote (Figure 4D; parameter *a* in Table 1)—reflect the drawback inherent in the non-destructive method of monitoring fluorescence when the tubercle tissue becomes more differentiated and less transparent. As a result, the measurement time is limited to the first week or so after attachment. In later developmental stages, when the parasite's phloem is formed and endogenous translocation of GFP is visible, the parasite tissues are even less transparent (Figure 1G).

Glyphosate that was applied during the independent phase charged the host plant with the herbicide, and the glyphosate therefore reached the parasite immediately after the connection had been established. As opposed to the observations on the untreated tubercles, in treated plants, *P. aegyptiaca* was unable to develop beyond the initial tubercle structure that was prevalent in the first 2–3 days after attachment (Figures 4A,C). In parallel, there was no substantial accumulation of GFP (Figure 4D), reflecting immediate injury to the tubercle tissue and inability of the tubercle to develop further.

#### Glyphosate inhibits GFP translocation to the parasite

**Figure 5:**
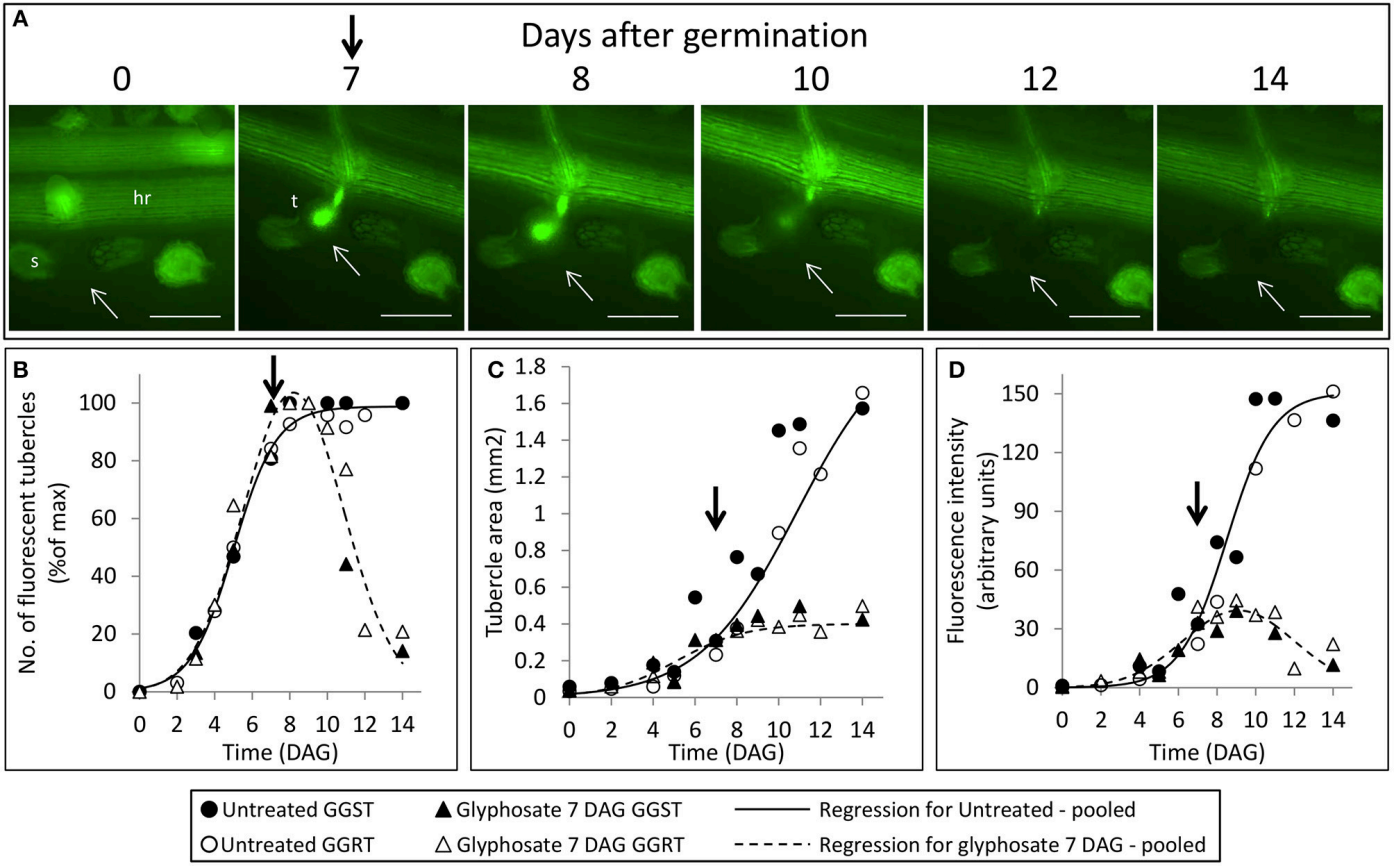
**Attachment and development of *P. aegyptiaca* following treatment with glyphosate at 7 DAG (post-attachment). (A)** Time course of attachment to GGRT following treatment with glyphosate, captured under a GFP3 filter. hr, host root; s, seedling; t, tubercle; Arrows indicate attachments. Bar length represents 0.5 mm. **(B)** Number of active attachments (represented by the number fluorescent tubercles), **(C)** area of the tubercle, and **(D)** accumulation of GFP in the tubercles (represented by the intensity of fluorescence) on GGST hosts (filled symbols) and on GGRT hosts (open symbols) following treatment with glyphosate at 7 DAG (triangles) or without treatment (circles). For non-linear regressions, data from GGST and GGRT hosts was subjected to *F*-test and combined. DAG—days after germination. Regression parameters are presented in Table 2. (*n* = 6). Vertical arrows indicate the time of glyphosate application (7 DAG).

During the parasitic phase, *P. aegyptiaca* relies on a continuous supply of nutrients from the host plant that is crucial for its growth and development. Therefore, it was important to clarify whether glyphosate has a secondary effect in inhibiting the translocation of solutes to the parasite and over what time frame. Despite the continuous translocation of GFP in GGRT roots—visible by its fluorescence (Figure 5A)—a darkening of the tubercles and cessation of GFP transport were observed for all the tubercles 3–4 days after “post-attachment” application of glyphosate (Figure 5A vs. Figure 3). This phenomenon was accompanied by the inability of the tubercles to develop and increase in area, as they did in the untreated control (Figure 5C). During the first 2 days after treatment, the fluorescence in the tubercles continued to increase, indicating a continuous accumulation of GFP, and maximal fluorescence was observed 9 DAG (Figure 5D). Nevertheless, this increase took place at lower rates compared with the accumulation rate in the untreated control, and the difference could be observed as early as 8 DAG, 24 HAT with glyphosate. This time frame was confirmed in another experiment with more frequent observations revealing that the levels of fluorescence intensity were stabilized as early as 24 HAT (data not shown). The results from both experiments indicate a deceleration or a complete inhibition of GFP movement to the parasite tubercles within a day after glyphosate application. In addition, it is important to note that the inhibition described above was similar for GGST and GGRT hosts, thereby demonstrating the specific influence of glyphosate solely on the parasite tissues.

#### Glyphosate alters the sugars profile of *P. aegyptiaca*

**Figure 6:**
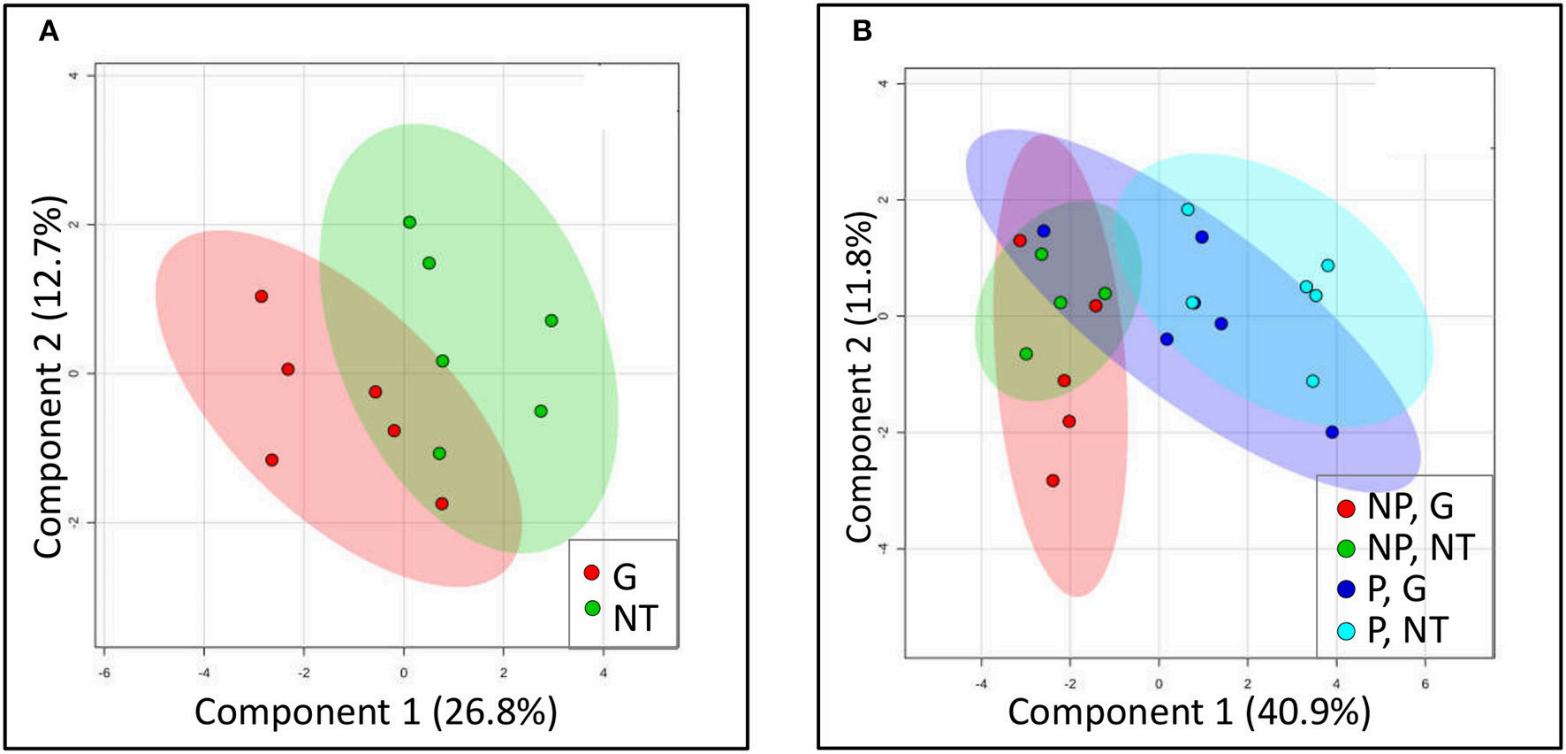
**Clustering of sugars profile using a partial least square–discriminant analysis (PLS-DA) model**. Models were constructed for **(A)** *P. aegyptiaca* under treatment with glyphosate (G) or without treatment (NT) and for **(B)** GRT roots comparing parasitized (P) or non-parasitized (NP) roots under treatment with glyphosate (G) or without treatment (NT). Each plotted point is a projection of the samples data on a two-dimensional plot. Colored area represents 95% confidence region. Co-variance explained by the two primary components is indicated in parentheses.

It is generally held that the reliance of *P. aegyptiaca* on the metabolism of certain carbohydrates allows it to attract more solutes from the vascular system of the host. In this part of the study, we sought an explanation for the inhibition of solutes translocation by glyphosate by examining changes in the sugars profile of *P. aegyptiaca*. To provide a graphic summary of the metabolomics data clustering patterns resulting from glyphosate treatment, a PLS-DA was computed. Profiles of sugars in *P. aegyptiaca* and GRT roots are presented in the score plots given in Figure 6. The analysis revealed a distinction between glyphosate-treated and untreated plants that clearly reflects the effect of glyphosate on the profile of the sugars metabolites in the parasite (Figure 6A). Although, there is no complete separation between the two groups, clustering could be observed. It appears that the sugars profile was less profoundly affected by the treatment in comparison with the total metabolites (data not shown), that included also the amino acids and other acids that participate in amino acids biosynthesis (Shilo et al., 2016). Naturally, the metabolites that are directly involved in the shikimate pathway and in amino acids biosynthetic pathways were separated into two distinct clusters (data not shown). These results strengthen our hypothesis that the herbicidal action of glyphosate includes alterations to the sugars profile in the parasite.

In the host roots, glyphosate treatment did not substantially change the metabolic profiles of sugars (Figure 6B). Parasitism, however, significantly altered this profile, as is apparent from their separate clustering (light blue, Figure 6B). It seems that the treatment of the parasitized host with glyphosate somewhat “pushed” the metabolic profile in the direction of the non-parasitized clusters, probably as a result of elimination of the parasites.

**Figure 7:**
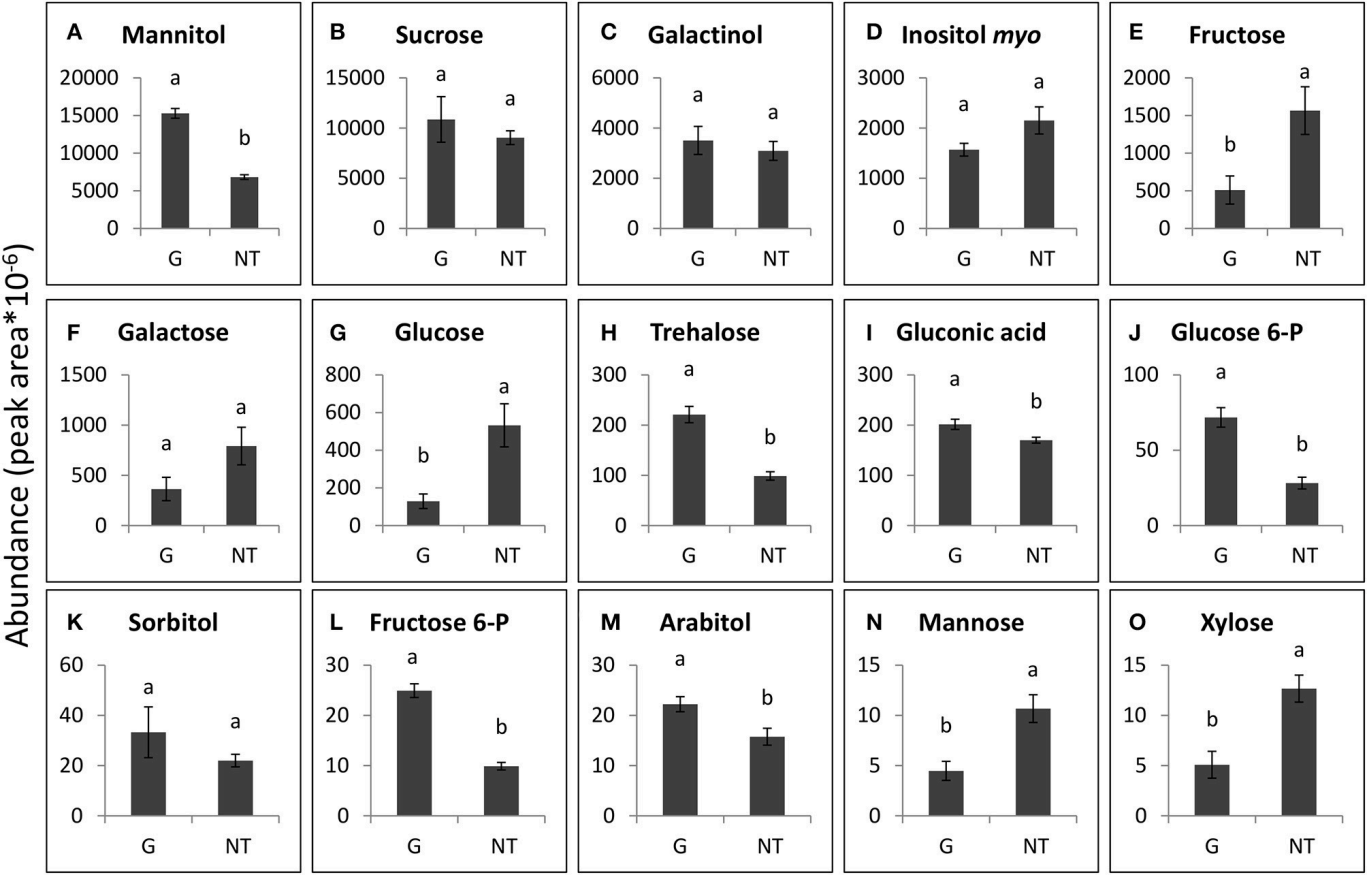
**Levels of the major sugars that were detected in *P. aegyptiaca* tubercles 48 h after treatment (HAT) with glyphosate (G) or untreated (NT)**. Graphs are arranged in descending abundance. Error bars represent standard error (*n* = 3). ANOVA was computed according to Student's *t*-test (*P* < 0.05). **(A)** Mannitol, **(B)** Sucrose, **(C)** Galactinol, **(D)** Inositol *myo*, **(E)** Fructose, **(F)** Galactose, **(G)** Glucose, **(H)** Trehalose, **(I)** Gluconic acid, **(J)** Glucose 6-P, **(K)** Sorbitol, **(L)** Fructose 6-P, **(M)** Arabitol, **(N)** Mannose, and **(O)** Xylose.

**Figure 8:**
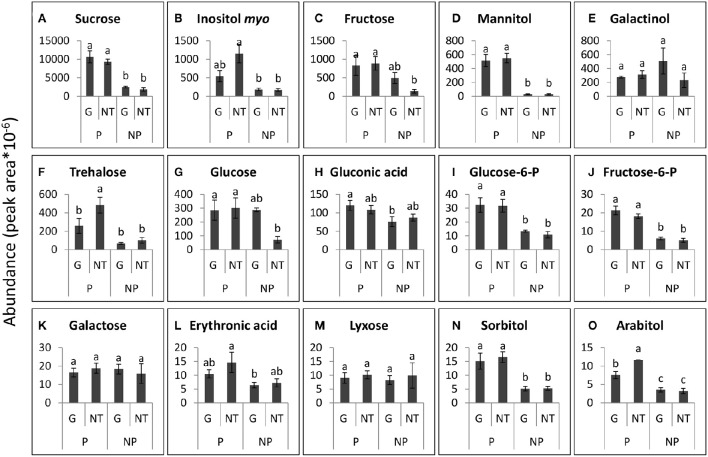
**Levels of the major sugars that were detected in the roots of GRT parasitized by *P*. *aegyptiaca* (P) or not parasitized (NP), 48 h after treatment (HAT) with glyphosate (G) or untreated (NT)**. Graphs are arranged in descending abundance. Error bars represent standard error (*n* = 3). ANOVA was computed according to Student's *t*-test (*P* < 0.05). **(A)** Sucrose, **(B)** Inositol *myo*, **(C)** Fructose, **(D)** Mannitol, **(E)** Galactinol, **(F)** Trehalose, **(G)** Glucose, **(H)** Gluconic acid, **(I)** Glucose-6-P, **(J)** Fructose-6-P, **(K)** Galactose, **(L)** Erythronic acid, **(M)** Lyxose, **(N)** Sorbitol, and **(O)** Arabitol.

The 15 soluble carbohydrates that were most abundant in *P. aegyptiaca* tubercles and in GRT roots are presented in Figures 7, 8. Mannitol and sucrose were found to be the most abundant sugars in the tubercles of *P. aegyptiaca* (Figures 7A,B). As implied in the score plot, treatment with glyphosate created a shift in most of the sugars that were detected in the parasite tubercles 48 HAT. Levels of the hexoses, fructose and glucose, were 3- and 4-fold lower than those detected in untreated control by 48 HAT, respectively (Figures 7E,G). In contrast, levels of other sugars, such as mannitol, glucose-6-P and fructose-6-P, exhibited an increase of approximately 2.5-fold at 48 HAT as compared with the untreated tubercles (Figures 7A,J,L). Sucrose levels did not differ significantly from the untreated control (Figure 7B).

In contrast to the findings in the tubercles, the levels of the sugars that were detected in the roots of the GRT host plants were not significantly altered by glyphosate treatment (Figure 8), confirming the strong resistance of the GRT host to glyphosate and indicating a specific influence of the herbicide on carbon metabolism in the parasite. Nonetheless, almost all sugars in the host root exhibited substantial increases in response to *P. aegyptiaca* parasitism. The most abundant sugars in the roots of parasitized GRT plants were sucrose, inositol-*myo*, fructose, mannitol, and galactinol (Figures 8A–E). Mannitol levels were almost undetectable in non-parasitized roots but soared to a level 20-fold higher than that in parasitized roots (Figure 8D). Sucrose levels increased by about 5-fold in response to the parasitism (Figure 8A), while inositol-*myo* and fructose increased by almost 7-fold when compared with non-parasitized roots (Figures 8B,C). Some sugars in parasitized roots, i.e., trehalose and arabitol, fell after treatment with glyphosate, probably as a result of the elimination of *P. aegyptiaca* (Figures 8F,O). Mannose and xylose were also detected in the roots of GRT plants but at lower levels and therefore are not presented in Figure 8.

Some of the sugars that were detected in *P. aegyptiaca* were notably elevated in comparison with levels in the adjacent host roots, indicating their accumulation or biosynthesis in the parasite. Among these sugars, mannitol was present in very high levels, 12-fold higher than those in the parasitized host roots (Figures 7A, 8D). Fructose and glucose levels were also higher in the tubercles than in roots of the host, with an almost 2-fold difference (Figures 7E,G, 8C,G). Other sugars that were also found to be substantially elevated in comparison with levels in the host roots were galactinol (Figures 7C, 8E) and galactose (Figures 7F, 8K), being10- and 42-fold higher, respectively. It is important to note that sucrose was not among the accumulating sugars in *P. aegyptiaca* tubercles, and its levels were largely unchanged compared to those in the adjacent host roots.

## Discussion

In the present study we demonstrated the secondary effects of glyphosate in controlling the holoparasite *P. aegyptiaca* in both of its life cycle phases—independent and parasitic.

### Effect of glyphosate on *P. aegyptiaca* during the independent phase

Glyphosate is usually applied to the plant foliage, from where it moves to the roots and sink tissues, mainly via the phloem (Gougler and Geiger, 1981; Schulz et al., 1990). In several species, it has been shown that glyphosate is exuded from the roots to the rhizosphere (Gomes et al., 2014). For example, glyphosate exudation was recorded from the roots of both soybean (*Glycine max*) and glyphosate-resistant soybean as early as 2 days after treatment (Kremer et al., 2005). In quinoa (*Chenopodium quinoa*), a residue of 4% of the applied glyphosate was detected in the adjacent soil 8 days after treatment (Laitinen et al., 2007). A similar phenomenon has been described in herbicides of the imidazolinones, whose exudation to the rhizosphere has been exploited as a weed control strategy against parasitic weeds (Kanampiu et al., 2002; Colquhoun et al., 2006; Eizenberg et al., 2013).

When simulating the effect of glyphosate on the parasite during the independent phase, we found that glyphosate did not inhibit the germination of *P. aegyptiaca* seeds or impair the potency of the seedlings (Figure 2), with the exception of the highest examined concentration of glyphosate (1,800 mg ae l^−1^) for which almost absolute inhibition of germination and seedling viability were observed (Figure 2). This dose is, however, unlikely to be found in the soil solution under *P. aegyptiaca* management practices (Cochavi et al., 2015, 2016). In addition, even if higher doses of glyphosate are applied, the seed is likely to encounter only an extremely small fraction of the initial herbicide dose in the soil because of the herbicide interaction with and adsorption to soil particles (Borggaard and Gimsing, 2008). The phenomenon of inhibition of germination by high concentrations of different substances has also been described by Whitney and Carsten (1981), who observed negative effects on germination and radicle elongation under high concentrations of germination stimulants. In those cases, the radicle may not even emerge from within the seed coat, making it appear as if the seeds have not germinated at all (Whitney and Carsten, 1981; Joel and Bar, 2013).

Lower doses of glyphosate actually had a positive effect on germination when the herbicide was applied after the germination process had begun (Figure 2B). The phenomenon of hormesis to herbicides is well documented and was reported in different plants in relation to glyphosate (Belz and Duke, 2014). In some *Orobanche* and *Phelipanche* species, similar findings have been reported for some growth regulators, such as herbicides of the group of phytoene synthase inhibitors, which had an additive effect in enhancing germination of the parasite when applied together with a germination stimulant (Chae et al., 2004; Song et al., 2005, 2006).

Seedlings of *P. aegyptiaca* were able to attach to both GGST and GGRT hosts roots even as long as 7 days after treatment with glyphosate (Figures 4A,B). This result further supports our hypothesis that glyphosate does not harm *P. aegyptiaca* seeds or seedlings during the independent phase of growth and that its herbicide action begins to take effect only after the parasite has established a connection to the vascular system of the host and begins to draw in glyphosate.

The establishment of a phloem connection by *P. aegyptiaca* to its host is a crucial step in the parasite survival, representing the transition from an independent organism incapable of long-term existence to a parasitic lifestyle (Joel, 2000). The progress of this developmental phase can be described by a non-linear regression (Figure 4B), indicating the important time points of the first established attachments and the range during which most connections take place in a population of *P. aegyptiaca* seeds.

In the GGST and GGRT plants, darkening (loss of fluorescence) of the glyphosate-treated attachments appeared 3–4 days after their connection to the host phloem (Figures 4A,B) as a result of translocation of the herbicide and the beginning of the mortality processes. Yet, it is reasonable to assume that injury to the parasite had begun to occur even earlier, possibly within hours after treatment, as the impairment of tubercle development was indeed visible at that time (Figures 4A,C). It has previously been shown, with fluorescein diacetate staining, that loss of viability in even larger tubercles of *P. aegyptiaca* was evident a few hours after treatment with glyphosate (Shilo et al., 2016). The early injury was specific to the haustorium region, which constituted most of the parasitic tissues in the young tubercle, as was the case in this study. In addition, the fluorescence in the tubercles might not exactly parallel the viability of the tissues because some time might be required for the GFP to be degraded or vacated to other tissues that will have become stronger sinks.

### Effect of glyphosate on *P. aegyptiaca* during the parasitic phase

As mentioned above, previous studies have shown that glyphosate translocates mainly in the phloem and readily reaches the sink tissues. For example, movement of [^14^C]glyphosate to *O. crenata* parasitizing garden peas (*Pisum sativum*) or faba beans (*Vicia faba*) or to *O. cumana* parasitizing sunflower (*Helianthus annuus*) plants reached a maximum 3 days after application (Arjona-Berral et al., 1990; Jain and Foy, 1997; Diaz-Sanchez et al., 2002). However, measurements of shikimate accumulation showed that the herbicide had already reached the tubercles a few hours after the application (Shilo et al., 2016). As we hypothesized, in our study of the tomato–*P. aegyptiaca* association, glyphosate had a rapid effect on translocation, limiting the parasite's ability to attract solutes via the phloem. According to the stabilization in fluorescence levels of GFP in the tubercles, it was evident that at least a deceleration, if not complete inhibition, of movement of GFP occurred within 24 HAT (Figure 5D). Due to this lack of synchronization between GFP fluorescence and the actual cessation of translocation of the protein, it is difficult to pinpoint exactly when the full inhibition of translocation occurred. Nonetheless, despite the macromolecular size of GFP, the movement of this protein in “GFP-mobile” plants is considered to provide a good simulation of solute translocation via the phloem and partitioning among different sink tissues (Imlau et al., 1999; Nadler-Hassar et al., 2004; Birschwilks et al., 2006). Thus, even a deceleration in GFP movement will provide an indication of general inhibition of solute translocation from the phloem. For example, translocation of GFP and [^14^C]sucrose to the shoot parasite *Cuscuta campestris* parasitizing tobacco plants was inhibited within 3 days of glyphosate application (Nadler-Hassar et al., 2004).

GGRT plants, which have the parental background of GRT plants, are not affected—symptomatically or biochemically—by the currently used dosages of glyphosate, as is apparent from their stable profiles of amino acids and other organic acids, including shikimate (Shilo et al., 2016). In addition, GGRT plants continued to distribute GFP throughout their vascular system, with their roots remaining fluorescent a week and even 2 weeks after glyphosate application (Figures 4A, 5A, respectively). These findings mean that the GGRT host continues to distribute solutes despite the treatment with glyphosate, as opposed to the GGST host that sustain an injury of its own. Nonetheless, the translocation pattern of GFP and its inhibition after treatment with glyphosate were similar in both glyphosate-sensitive and glyphosate-resistant hosts parasitized by *P. aegyptiaca*. The importance of this finding lies in its contribution to differentiating between the effects of glyphosate on the parasite and those on the host plants, indicating that it is the parasite that is independently responsible for the extent of translocation in the host-parasite system.

In addition, it was found that glyphosate created a shift in the sugars profile of *P. aegyptiaca*, measured at 48 HAT (Figures 6A, 7) but had little effect on the sugars profile in the host roots, with or without parasite (Figures 6B, 8). This finding implies that the changes in the sugars profile are not a result of inhibition of the translocation of sugars but are more likely to be the result of disruption of metabolism in the parasite tissues. Apparently, metabolites participating in the pathways closest to the shikimate pathway will be affected before the sugars. About 30% of the assimilated carbon in plants enter the shikimate pathway (Maeda and Dudareva, 2012). If EPSPS is inhibited, a large portion of this carbon accumulates in the form of shikimate, preventing the metabolism of carbon assimilates by other important pathways. It was previously found that the accumulation of shikimate in the parasite in the tomato–*P. aegyptiaca* system started at 10 HAT (Shilo et al., 2016), but it probably takes time for the parasite to accumulate the substantial levels of shikimate that will force alterations to other important carbon metabolic pathways. In *P. aegyptiaca*, some carbon metabolic pathways, e.g., that of mannitol, are unique to this family of root parasites and are thought to be of crucial importance to its ability to attract solutes and successfully compete with the sinks of the host (Westwood, 2013). Therefore, an alteration of the carbon metabolism in *P. aegyptiaca* might provide a possible explanation for the weakening of its sink strength, actually preventing it from maintaining the driving force necessary for the attraction of solutes. It has previously been reported that glyphosate also interrupts, within hours of application, carbon metabolism in other plant species and in other organisms. For instance, glyphosate disrupted C_3_ cycle metabolism in source leaves of sugar beet plants a few hours after application, inducing limited translocation throughout the plant (Geiger et al., 1999). In velvetleaf (*Abutilon theophrasti*), glyphosate had a more destructive effect on sink tissues because of its rapid movement to the root system (Fuchs et al., 2002). In both leaves and roots of pea plants, changes in levels of sugars were observed in response to glyphosate application, as manifested by an accumulation of sucrose, hexose and starch 1 day after treatment (Orcaray et al., 2012). In addition, changes in levels of transcripts related to the glycolysis process were reported in soybean as early as four HAT with glyphosate (Zhu et al., 2008). Nonetheless, based on our results, we are not able to attribute the alterations in sugars profile to a specific event occurring as a result of the inhibition of EPSPS and the shikimate pathway. Further investigation is required to determine the exact pathways in *P. aegyptiaca* that are influenced following EPSPS inhibition.

Tubercles of *P. aegyptiaca* displayed elevated amounts of mannitol (Figure 7A) and alterations in all other sugars (namely, fructose, glucose, mannose, glucose-6-P and fructose-6-P) that participate in mannitol biosynthesis 48 HAT with glyphosate (Figure 7; Harloff and Wegmann, 1993). Mannitol is known to serve as an osmoregulant in algae and fungi, and, as mentioned above, its biosynthetic pathway is crucial to the *P. aegyptiaca* and its holoparasite relatives (Harloff and Wegmann, 1993; Aly et al., 2009). In addition, the levels of fructose and glucose—the products of sucrose cleavage that enable its continuous transport (Draie et al., 2011; Fernández-Aparicio et al., 2016)—were significantly decreased in the tubercles in response to glyphosate treatment (Figure 7). In contrast, the levels of sucrose in *P. aegyptiaca* tubercles were similar to its levels in the adjacent tomato roots (Figures 7B, 8A). This finding was surprising, since one would expect that the inhibition of translocation would be reflected in reduced levels of translocated sucrose. However, it is possible that simultaneous synthesis of sucrose might take place, as becomes apparent from the reduction of its products. has been documented in nodules of nodulated lupine (*Lupinus albus*) plants treated with glyphosate in which there was elevated activity of sucrose synthase concomitant with increased sucrose levels and a reduction in starch production (De María et al., 2006).

The fact that glyphosate induced little effect on the profile of sugars in GRT roots (Figures 6E, 8) further supports our supposition of the high resistance of the GRT host to glyphosate. On the other hand, the parasitism *per se* had a strong effect on the amounts of sugars that were abundant in the host roots (Figures 6E, 8). In other cases of root parasitism, metabolomics changes were stimulated in the tissues of the host plant. For example, in the root hemi-parasite *Striga hermontica* parasitizing sorghum (*Sorghum bicolor*) and in *O. foetida* parasitizing faba beans certain changes in the metabolomic profiles were observed in the xylem sap and leaf phloem of these plants, respectively (Pageau et al., 2003; Abbes et al., 2009a). In contrast, Hacham et al. (2016) found that parasitism of *P. aegyptiaca* had no profound effect on metabolic profile of the tomato host, inducing elevation of only 8 of a total of 59 metabolites that were detected in its roots, although some of those metabolites were carbohydrates. It is important to remember that sometimes even different phenological stages, let alone different experimental systems and different environmental conditions, could affect the metabolomics profile and the translocation profile in the host-parasite system (Westwood, 2013) and might provide an explanation for the above differences.

## Conclusions

Holoparasites differ from autotrophic plants in that they depend on photosynthesis and carbon assimilation of another organism. Nonetheless, it seems that *P. aegyptiaca* does rely on its own carbon metabolism to provide the driving force for the attraction of solutes. In the current study, it was shown that glyphosate has a secondary mechanism of action in *P. aegyptiaca*, acting via inhibition of the translocation of large molecules (e.g., GFP) and probably of other phloem solutes as well. Although, the detailed chain of events is not yet clear, it appears that glyphosate's alteration of the parasite's sugars profile after EPSPS inhibition might provide an explanation for the decreased ability of *P. aegyptiaca* to attract solutes via the phloem. In addition, it was shown that glyphosate affects *P. aegyptiaca* only post attachment to the host root and does not seem to affect parasite seed germination and establishment on the host root.

## Author contributions

TS, BR, SW, and HE conceived and designed research. TS conducted experiments. DP, SG, and YH assisted with experiments execution. RA assisted with data analysis. TS analyzed data and wrote the manuscript. All authors read and approved the manuscript.

### Conflict of interest statement

The authors declare that the research was conducted in the absence of any commercial or financial relationships that could be construed as a potential conflict of interest.
